# Serum iron status and the risk of breast cancer in the European population: a two-sample Mendelian randomisation study

**DOI:** 10.1186/s12263-021-00691-7

**Published:** 2021-07-06

**Authors:** Chenyang Hou, Qingzhi Hou, Xing Xie, Huifeng Wang, Yueliang Chen, Tingxi Lu, Qunying Wu, Yongcong Liang, Yanling Hu, Yuang Mao

**Affiliations:** 1 Department of Information and Management, Guangxi Medical University, Nanning, 530000 Guangxi China; 2 Department of Occupational Health and Environmental Health, School of Public Health, Shandong First Medical University (Shandong Academy of Medical Sciences), Taian, 271000 Shandong China; 3 Life Sciences Institute, Guangxi Medical University, Nanning, 530000 Guangxi China; 4 Department of Biochemistry and Molecular Biology, School of Pre-Clinical Medicine, Guangxi Medical University, Nanning, 530000 Guangxi China

**Keywords:** Iron status, Breast cancer, ER-positive breast cancer, ER-negative breast cancer, Mendelian randomisation

## Abstract

**Background:**

Previous observational studies have provided conflicting results on the association between serum iron status and the risk of breast cancer. Considering the relevance of this relationship to breast cancer prevention, its elucidation is warranted.

**Object:**

We used a two-sample Mendelian randomisation (MR) study to explore the causal relationship between serum iron status and the risk of breast cancer.

**Method:**

To select single nucleotide polymorphisms (SNPs) that could be used as instrumental variables for iron status, we used the Genetics of Iron Status consortium, which includes 11 discovery and 8 replication cohorts, encompassing 48,972 individuals of European descent. Moreover, we used the OncoArray network to select SNPs that could be considered instrumental variables for the outcome of interest (breast cancer); this dataset included 122,977 individuals of European descent with breast cancer and 105,974 peers without breast cancer. Both conservative (SNPs associated with overall iron status markers) and liberal (SNPs associated with the levels of at least one iron status marker) approaches were used as part of the MR analysis. For the former, we used an inverse-variance weighted (IVW) method, whereas for the latter, we used the IVW, MR-Egger regression, weighted median and simple mode methods.

**Results:**

When the conservative approach was used, iron status showed no significant association with the risk of breast cancer or any of its subtypes. However, when the liberal approach was used, transferrin levels were found to be positively associated with the risk of ER-negative breast cancer based on the simple mode method (OR for MR, 1.225; 95% CI, 1.064, 1.410; *P* = 0.030). Nevertheless, the levels of the other iron status markers showed no association with the risk of breast cancer or its subtypes (*P* > 0.05).

**Conclusion:**

In our MR study, the liberal approach suggested that changes in the concentration of transferrin could increase the risk of ER-negative breast cancer, although the levels of other iron status markers had no effect on the risk of breast cancer or its subtypes. This should be verified in future studies.

**Supplementary Information:**

The online version contains supplementary material available at 10.1186/s12263-021-00691-7.

## Introduction

The morbidity of breast cancer is increasing, affecting the quality of life of patients and their families and increasing their economic burden. Oxidative stress, a known cause of breast cancer, may have a role in this process [1]. Iron is a necessary micronutrient for the human body [2], and it plays an important role in various physiological processes, such as electron transfer, oxygen transport, immune function, DNA synthesis and energy production [3, 4]. Moreover, iron catalyses the generation of reactive oxygen species, and it could thus increase both oxidative stress and oncogene activation. Therefore, levels of iron in the body may affect the development of breast cancer [2, 5, 6].

Previous epidemiological studies have reported that higher levels of iron may be associated with a modestly increased risk of breast cancer [7, 8]. In contrast, studies by Quintana Pacheco et al. and Cade et al. have found that high levels of iron show an inverse relationship with breast cancer risk [9, 10]. Further, study by Kabat et al. has shown that iron status is not associated with the risk of breast cancer [11]. The link between iron status and breast cancer risk is therefore under much debate.

Previous studies have reported the presence of confounding factors, such as post-natal living environment, behaviour and habits, social status and environmental factors, that affect the association between iron status and breast cancer risk [12]. Indeed, such confounding factors could affect the causal inferences obtained in traditional epidemiological studies. Moreover, it is possible that breast cancer and iron levels have a reverse causal relationship, which could further affect the conclusions of traditional observational epidemiological studies.

Mendelian randomisation (MR) — a process in which genetic variations closely related to the exposure variable are used as instrumental variables — can help overcome the limitations of traditional epidemiological studies and allow us to make causal inferences regarding the effect of a particular exposure on an outcome [13]. Because alleles follow random distribution during gametogenesis, fertilised eggs have random genetic variants; thus, the genetic variations associated with the outcome or exposure are not affected by confounding factors or reverse causality [12].

To our knowledge, there have been no MR-based studies examining the relationship between iron status and breast cancer risk, even though the delineation of this relationship could have significant utility in breast cancer prevention and treatment. To this end, in the present study, we used MR to investigate whether iron status is related to the incidence of breast cancer using publicly available data from genome-wide association studies (GWASs).

## Methods

**Fig. 1:**
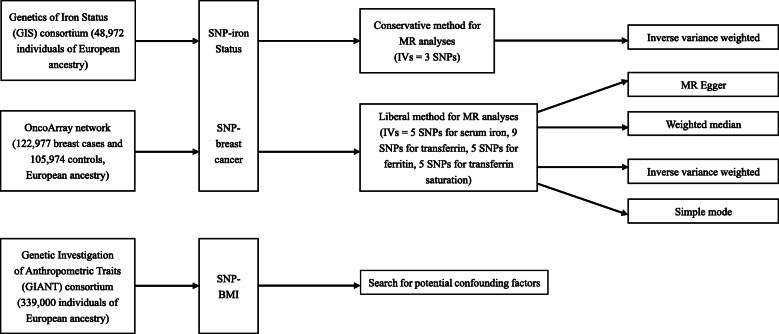
Related databases and analysis methods in MR analysis. The publicly available summary data of SNP phenotypes were obtained from the largest meta-GWAS databases. The effect of iron status on asthma was estimated using a conservative approach (IVs: only SNPs connected with the concentrations of ferritin, serum iron and transferrin saturation were increased, concentrations of transferrin was decreased and systemic iron status would be increased) and a liberal approach (IVs: one of the SNPs was associated with breast cancer). In conservative instruments set, the inverse-variance-weighted (IVW) method was used, and in liberal instruments set, the IVW, MR Egger regression, weighted median and simple mode methods were used. MR, Mendelian randomization; IVs, instrumental variables; SNP, single nucleotide polymorphism; MR Egger, Mendelian randomization–Egger regression method; BMI, body mass index

We applied two-sample MR to summary data from the respective GWASs (Fig. 1). The original studies had been performed after obtaining informed consent from participants and had also received ethical approval. We used single nucleotide polymorphisms (SNPs) that showed strong relationships with total serum iron status as instrumental variables to explore the effect of iron status on breast cancer risk. By adjusting for the relationship of the instrumental variables (SNPs) with iron status and breast cancer, we estimated the effect of systemic iron status on breast cancer risk. To investigate whether iron status acts as a potential bias or mediator in the relationship between breast cancer risk and other breast cancer risk factors, we analysed the relationship between the SNPs associated with iron status and breast cancer risk factors.

### Data sources: exposure

We obtained summary data from the largest meta-GWAS containing information on iron status, the Genetics of Iron Status (GIS) consortium, and performed MR analysis. This database included data on serum iron levels, transferrin levels, ferritin levels, and transferrin saturation. Data from 11 discovery and 8 replication cohorts encompassing 48,972 individuals of European ancestry were collected [14]. Benyamin et al. had performed genome-wide analyses within each cohort based on a uniform analysis plan after adjustment for factors such as principal component scores and age. Furthermore, the thresholds of the population stratification and quality control for each cohort were imputed into the score > 0.5, Hardy–Weinberg Equilibrium (HWE) ≥ 10^-6^, minor allele frequency (MAF) > 0.01 [14].

### Data sources: outcome

Publicly available summary data on breast cancer were obtained from the largest meta-GWAS containing data on breast cancer risk — the OncoArray network — which contains data regarding five cancers: breast, ovarian, prostate, lung, and colorectal cancer [15]. The breast cancer database contained information on 122,977 individuals of European ancestry with breast cancer (cases) and 105,974 peers (controls). Of the case cohort, 69,501 individuals had ER-positive and 21,468 individuals had ER-negative breast cancer.

The thresholds of the population stratification and quality control for the GWAS analysis for the cohort were imputed into the score > 0.3, HWE ≥ 10^−12^, and MAF > 0.01. This information has been previously reported [16, 17].

### Selection of instrumental variables

Instrumental variables that showed a strong association with iron status (*P* < 5 × 10^−8^) were selected from the GIS consortium (Tables 1, 2, 3, 4) [14]. All the SNPs could be found in the OncoArray network and showed linkage equilibrium (all pairwise r^2^ ≤ 0.01). None of the SNPs that were chosen as instrumental variables were correlated with breast cancer (*P* > 0.05). The corresponding statistical indicators (beta and SE) were obtained from the GIS consortium and OncoArray network databases. SNPs for iron status from the GIS consortium corresponded to the European population, with a sample size of 48,972 cases. SNPs for breast cancer from the OncoArray network database corresponded to the same European ethnic group, with a sample size of 122,977.

**Table 1:** Effect of iron concentration GWAS identified variants on breast cancer

SNPs	Chr: BP (Build 37)	Locus	Effect/other allele	Iron (μmol/L)		Overall breast cancer		ER-positive breast cancer		ER-negative breast cancer	
				Beta^b^ (SE)	*P* value	Beta^c^ (SE)	*P* value	Beta^c^ (SE)	*P* value	Beta^c^ (SE)	*P* value
rs8177240	3: 133,477,701	*TF*	T/G	−0.066(0.007)	6.65E−20	−0.014 (0.007)	0.040	−0.014 (0.008)	0.072	−0.012 (0.012)	0.304
rs1800562^a^	6: 26,093,141	*HFE*	A/G	0.328(0.016)	2.72E−97	−0.003 (0.014)	0.831	0.010 (0.016)	0.535	−0.027 (0.025)	0.288
rs7385804	7: 100,235,970	*TFR2*	A/C	0.064(0.007)	1.36E−18	0.016 (0.007)	0.015	0.010 (0.008)	0.225	0.017 (0.012)	0.151
rs855791^a^	22: 37,462,936	*TMPRSS6*	A/G	−0.181(0.007)	1.32E−139	−0.003 (0.006)	0.627	0.001 (0.008)	0.852	−0.013 (0.012)	0.255
rs1799945^a^	6: 26,091,179	*HFE*	C/G	−0.189(0.010)	1.10E−81	−0.001 (0.009)	0.913	−0.003 (0.011)	0.804	−0.014 (0.016)	0.380

^a^3 SNPs used in the conservative analyses. *GWAS* genome-wide association studies, *SNPs* single nucleotide polymorphisms, *Chr* chromosome, *BP* base pair, *SE* standard error, *ER* oestrogen receptor

^b^Beta units are per-allele effect estimates in iron concentrations

^c^Per-allele logarithm of the odds ratios between breast cancer cases and controls

**Table 2:** Effect of transferrin concentration GWAS identified variants on breast cancer

SNPs	Chr: BP (Build 37)	Locus	Effect/other allele	Transferrin (μmol/L)		Overall breast cancer		ER-positive breast cancer		ER-negative breast cancer	
				Beta^b^ (SE)	*P* value	Beta^c^ (SE)	*P* value	Beta^c^ (SE)	*P* value	Beta^c^ (SE)	P value
rs744653	2: 190,378,750	*WDR75–SLC40A1*	T/C	0.068 (0.010)	1.35E−11	−0.009 (0.009)	0.3548	−0.007 (0.011)	0.500	−0.016 (0.016)	0.310
rs8177240	3: 133,477,701	*TF*	T/G	−0.380 (0.007)	8.43E−610	−0.014 (0.007)	0.039	−0.014 (0.008)	0.072	−0.012 (0.012)	0.304
rs9990333	3: 195,827,205	*TFRC*	T/C	−0.051 (0.007)	1.95E−13	−0.012 (0.007)	0.059	−0.015 (0.008)	0.062	−0.015 (0.012)	0.210
rs1800562^a^	6: 26,093,141	*HFE*	A/G	−0.479 (0.016)	8.90E−196	−0.003 (0.014)	0.831	0.010 (0.016)	0.535	−0.027 (0.025)	0.288
rs1799945^a^	6: 26,091,179	*HFE*	C/G	0.114 (0.010)	9.36E−30	−0.001 (0.009)	0.913	−0.003 (0.011)	0.804	−0.014 (0.016)	0.380
rs4921915	8: 18,272,466	*NAT2*	A/G	0.079 (0.009)	7.05E−19	−0.010 (0.007)	0.187	−0.012 (0.009)	0.186	−0.014 (0.013)	0.295
rs174577	11: 61,604,814	*FADS2*	A/C	0.062 (0.007)	2.28E−17	−0.006 (0.007)	0.341	0.002 (0.008)	0.761	−0.033 (0.012)	0.007
rs6486121	11: 13,355,770	*ARNTL*	T/C	−0.046 (0.007)	3.89E−10	−0.004 (0.006)	0.587	−0.002 (0.007)	0.809	−0.009 (0.012)	0.450
rs855791^a^	22: 37,462,936	*TMPRSS6*	A/G	0.044 (0.007)	1.98E−09	−0.003 (0.006)	0.627	0.001 (0.008)	0.852	−0.013 (0.012)	0.255

^a^3 SNPs used in the conservative analyses. *GWAS* genome-wide association studies, *SNPs* single nucleotide polymorphisms, *Chr* chromosome, *BP* base pair, *SE* standard error, *ER* oestrogen receptor

^b^Beta units are per-allele effect estimates in transferrin concentrations

^c^Per-allele logarithm of the odds ratios between breast cancer cases and controls

**Table 3:** Effect of ferritin concentration GWAS identified variants on breast cancer

SNPs	Chr: BP (Build 37)	Locus	Effect/other allele	Ferritin (log) (μmol/L)		Overall breast cancer		ER-positive breast cancer		ER-negative breast cancer	
				Beta^b^ (SE)	*P* value	Beta^c^ (SE)	*P* value	Beta^c^ (SE)	*P* value	Beta^c^ (SE)	*P* value
rs744653	2: 190,378,750	*WDR75–SLC40A1*	T/C	−0.089 (0.010)	8.37E−19	−0.008 (0.009)	0.355	−0.007 (0.011)	0.500	−0.016 (0.016)	0.310
rs1800562^a^	6: 26,093,141	*HFE*	A/G	0.204 (0.016)	1.54E−38	−0.003 (0.014)	0.831	0.010 (0.016)	0.535	−0.027 (0.025)	0.288
rs1799945^a^	6: 26,091,179	*HFE*	C/G	−0.065 (0.010)	1.71E−10	−0.001 (0.009)	0.913	−0.003 (0.011)	0.804	−0.014 (0.016)	0.380
rs411988	17: 56,709,034	*TEX14*	A/G	−0.044 (0.007)	1.59E−10	−0.019 (0.006)	0.003	−0.014 (0.008)	0.079	−0.027 (0.012)	0.020
rs855791^a^	22: 37,462,936	*TMPRSS6*	A/G	−0.055 (0.007)	1.98E−09	−0.003 (0.006)	0.627	0.001 (0.008)	0.852	−0.013 (0.012)	0.255

^a^3 SNPs used in the conservative analyses. *GWAS* genome-wide association studies, *SNPs* single nucleotide polymorphisms, *Chr* chromosome, *BP* base pair, *SE* standard error, *ER* oestrogen receptor

^b^Beta units are per-allele effect estimates ferritin concentrations

^c^Per-allele logarithm of the odds ratios between breast cancer cases and controls

**Table 4:** Effect of transferrin saturation concentration GWAS identified variants on breast cancer

SNPs	Chr: BP (Build 37)	Locus	Effect/other allele	Transferrin saturation (μmol/L)		Overall breast cancer		ER-positive breast cancer		ER-negative breast cancer	
				Beta^b^ (SE)	*P* value	Beta^c^ (SE)	*P* value	Beta^c^ (SE)	*P* value	Beta^c^ (SE)	*P* value
rs8177240	3: 133,477,701	*TF*	T/G	0.100 (0.008)	7.24E−38	−0.014 (0.007)	0.039	−0.014 (0.008)	0.072	−0.012 (0.012)	0.304
rs1800562^a^	6: 26,093,141	*HFE*	A/G	0.577 (0.016)	2.19E−270	−0.003 (0.014)	0.831	0.010 (0.016)	0.535	−0.027 (0.025)	0.288
rs1799945^a^	6: 26,091,179	*HFE*	C/G	−0.231 (0.010)	5.13E−109	−0.001 (0.009)	0.913	−0.003 (0.011)	0.804	−0.014 (0.016)	0.380
rs7385804	7: 100,235,970	*TFR2*	A/C	0.054 (0.008)	6.07E−12	0.016 (0.007)	0.015	0.010 (0.008)	0.225	0.017 (0.012)	0.151
rs855791^a^	22: 37,462,936	*TMPRSS6*	A/G	−0.190 (0.008)	6.41E−137	−0.003 (0.006)	0.627	0.001 (0.008)	0.852	−0.013 (0.012)	0.255

^a^3 SNPs used in the conservative analyses. *GWAS* genome-wide association studies, *SNPs* single nucleotide polymorphisms, *Chr* chromosome, *BP* base pair, *SE* standard error, *ER* oestrogen receptor

^b^Beta units are per-allele effect estimates in saturation concentration concentrations

^c^Per-allele logarithm of the odds ratios between breast cancer cases and controls

To select instrumental variables, we used two analysis methods: conservative and liberal variable analyses. For conservative variable analyses, 3 SNPs (*rs855791*, *rs1800562*, *rs1799945*) strongly associated with the increasing concentrations of ferritin, serum iron and transferrin saturation and decreasing concentrations of transferrin (*P* < 5 × 10^−8^) were selected. Increased concentrations of ferritin and serum iron; increased transferrin saturation and decreased concentrations of transferrin would cause an improvement in systemic iron status [18]. Therefore, the genetic instrumental variables would have a coincident relationship with iron status via these four markers.

For liberal variable analyses, the SNPs strongly affiliated with at least one of the iron markers (5 SNPs for serum iron, 9 SNPs for transferrin, 5 SNPs for ferritin and 5 SNPs for transferrin saturation) in the GWAS (*P* < 5 × 10^−8^) were selected. Previous study has shown that there is a causal relationship between body mass index (BMI) and breast cancer [19]. Therefore, in order to rule out the possible confounding effect on the relationship between iron status and breast cancer, SNPs associated with BMI were retrieved from the GWAS database, GIANT, and we verified whether any of these were selected as instrumental variables in our study. In addition, we ensured that none of the instrumental variables were associated with the risk of breast cancer (all *P* > 0.05).

### Validation of selected instrumental variables

**Fig. 2:**
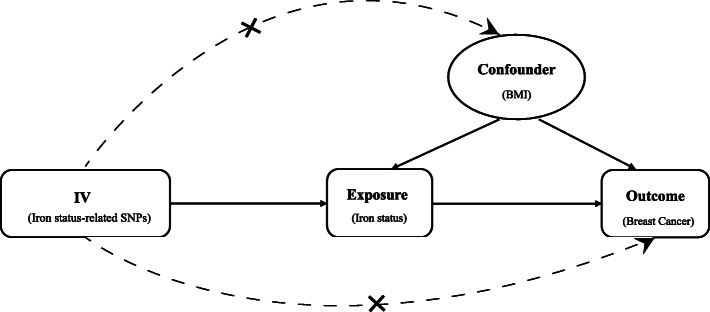
Diagram of the hypothesis of instrumental variables in Mendelian randomization study. IV, instrumental variable; BMI, body mass index

To be validated as instrumental variables, the selected SNPs had to meet three important criteria [20]. First, they had to be strongly associated with the exposure (iron status). Second, they had to not be associated with any confounders of the relationship between the exposure (iron status) and outcome (breast cancer). Finally, they should have influenced the outcome (breast cancer) only via the exposure (iron status) (Fig. 2).

To minimise any possible weak instrumental variable bias, we ensured that the F statistic was above 10, indicating that the study had sufficient strength. We ensured that the F statistic for the instrumental variables was above 10, and this was used to impose restrictions on the possible bias [21]. To limit the possibility of bias related to population stratification, we ensured that both exposure and outcome cohorts included individuals of European descent. We performed three analyses to resolve issues associated with pleiotropy. First, we assessed the SNPs that were known asthma risk factors associated with breast cancer. Second, to analyse MR estimates, we used two approaches — the conservative approach (primary analysis) and the liberal approach (secondary analysis). Third, unknown directional pleiotropy was assessed using the MR-Egger test.

### MR analyses

Two-sample MR was performed for testing the causal relationship between iron status and breast cancer. Moreover, breast cancer was subdivided as ER-positive and ER-negative breast cancer. Conservative and liberal methods were used in the MR analyses. As part of the conservative analysis, we used the inverse-variance-weighted (IVW) [21] method to conduct MR analyses. As part of the liberal analysis, we used the IVW, MR-Egger regression [22], weighted median, and simple mode methods [23] to estimate the effect of iron status on breast cancer risk. All of the data were selected from the OncoArray network and GIS consortium which were publicly available GWAS data (Fig. 1). R package ‘TwoSampleMR’ was used for MR analysis. Beta, SE and *P* values were obtained for the MR analysis. Beta values were transformed to odds ratios (ORs) using the following formula: OR = exp (beta). The 95% confidence interval (CI) was computed as follows: CI = exp (beta ± 1.96 × SE). Due to the limited consistency in publicly available data, the relationship between instrumental variables and other potential confounders, such as exercise and drinking, was difficult to assess. Hence, we used the GWAS catalogue database (https://www.ebi.ac.uk/gwas) to search for other phenotypes (including exercise, drinking, smoking, C-reactive protein levels, white blood cell counts) related to the selected instrumental variable SNPs and manually removed these SNPs from the MR analysis to rule out possible pleiotropic effects.

### Sensitivity analyses

For the sensitivity analyses, we used the IVW and MR-Egger tests to evaluate heterogeneity and displayed the results using forest plots showing the value for each SNP and Cochran’s Q statistics [24, 25]. In addition, by deleting one SNP at a time and recomputing estimates for the overall instrument variable pool, a leave-one-out analysis was performed to identify SNPs that had a greater or non-proportional effect. To ensure that the MR analysis results were more robust, we also performed an MR-Egger statistical sensitivity analysis, which limited the pleiotropic effects of the instrumental variables. In MR-Egger regression, the intercept, as an indicator of the average pleiotropic deviation, is allowed to be freely estimated [26]. In the conservative approach, the MR-Egger method regression was not performed to test pleiotropic effects, because only 3 SNPs were used and one SNP was removed for LD, making the data insufficient for analysis [27]. For the same reason, the pleiotropic effects of iron and transferrin saturation on overall breast cancer risk could not be tested.

All above analyses were performed by R, version 3.6.1.

## Results

### Instrumental variables for iron status and breast cancer risk

Tables 1, 2, 3 and 4 show the connections of iron status with SNPs that were used as instrumental variables in the liberal and conservative analyses. For the liberal analyses, Tables 1, 2, 3 and 4 show the characteristics of the genetic variation related to iron concentration (3 SNPs for overall breast cancer and 5 SNPs for ER-positive breast cancer and ER-negative breast cancer), transferrin concentration (9 SNPs for ER-positive breast cancer and 8 SNPs for overall breast cancer and ER-negative breast cancer), ferritin (log) concentration (4 SNPs for ER-positive breast cancer and 5 SNPs for overall breast cancer and ER-negative breast cancer) and transferrin saturation concentration (3 SNPs for overall breast cancer and 5 SNPs for ER-positive breast cancer and ER-negative breast cancer), respectively. For the conservative analyses, we used 3 SNPs association with iron levels, transferrin levels, ferritin levels and transferrin saturation (*rs1800562*, *rs1799945*, and *rs855791*). F statistics for all the instrumental variables ranged from 40 (*rs651007*, *ABO* gene) to 3346 (*rs8177240*, *TF* gene), showing that all the SNPs were strong instrumental variables (Tables 1, 2, 3, 4).

### The genetic instrument and breast cancer risk

None of the individual SNPs were associated with BMI, which is a confounding factor for breast cancer. In short, none of the selected variables associated with breast cancer risk in the liberal or conservative analyses were associated with increasing BMI (all *P* > 0.05) (Table 5).

**Table 5:** *P* value for the relationship between BMI and all genetic variations

SNPs	BMI (kg/m^**2**^)^a^ *P*
rs174577	0.104
rs1799945	0.124
rs1800562	0.124
rs411988	0.236
rs4921915	0.236
rs6486121	0.078
rs7385804	0.502
rs744653	0.813
rs8177240	0.941
rs855791	0.083
rs9990333	0.937

^a^*P* value for relationship between SNPs and BMI selected from the Genetic Investigation of Anthropometric Traits consortium

### Effect of iron status on breast cancer

**Fig. 3:**
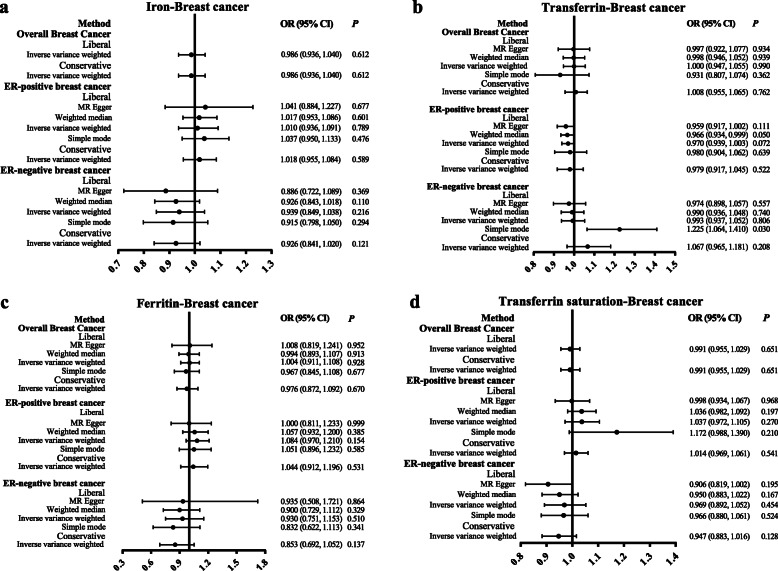
MR estimates the association of genetically predicted iron status and the risk of breast cancer. The results showed ORs for breast cancer and their subtype per SD increase in every iron status biomarker. **a**–**d** for iron, transferrin, ferritin, and transferrin saturation. MR, Mendelian randomization; MR-Egger, Mendelian randomization–Egger regression method; OR: odds ratio; 95% CI, 95% confidence interval; ER, oestrogen receptor

Figure 3 shows the results of MR in examining the association of genetically predicted iron status with the risk of breast cancer. The ORs for the risk of breast cancer and its subtypes per SD increase in the levels of each iron marker are displayed. In the conservative analysis, none of the four iron status was found to be associated with overall breast cancer, ER-positive breast cancer and ER-negative breast cancer risk (all *P* > 0.05). In the liberal analysis, we found a positive correlation between transferrin levels and ER-negative breast cancer risk based on the simple mode (OR, 1.225; 95% CI, 1.064, 1.410; *P* = 0.030). However, the status of other iron markers had no association with the risk of breast cancer or its subtypes (*P* > 0.05).

### Sensitivity analyses

For MR estimates, we used the liberal instruments method, and the heterogeneity for overall breast cancer, ER-positive breast cancer, and ER-negative breast cancer did not show statistical significance (all *P* > 0.05). Using the IVW method, we found no evidence of heterogeneity for the associations between the status of the four iron markers (iron, transferrin, ferritin and transferrin saturation) and breast cancer risk (for overall breast cancer: Q 0.02, 7.59, 1.12 and 0.08; for ER-positive breast cancer: Q 4.99, 6.21, 2.07 and 3.16; for ER-negative breast cancer: Q 3.65, 7.68, 2.81 and 4.66, respectively; all *P* > 0.05). Moreover, when using the MR-Egger method in the liberal analysis, we did not identify aggregated directional pleiotropy between the levels of the four iron markers and breast cancer risk (ER-positive breast cancer: intercept −0.005, 0.003, 0.008 and 0.012; ER-negative breast cancer: intercept 0.010, 0.005, −0.001 and 0.017; overall breast cancer: intercept 0.0005 [transferrin] and −0.0004 [ferritin]; all *P* > 0.05) (Supplementary Figs. 1, 2 and 3).

Importantly, the MR estimates did not radically change when the leave-one-out analyses were performed, although the estimated direction showed differences (Supplementary Figs. 4, 5, 6 and 7). Previous GWASs have reported that *rs174577* is also related with low-density lipoprotein cholesterol (LDL-D), high-density lipoprotein cholesterol (HDL-C), triglyceride (TG) and total cholesterol (TCHO) levels; *rs4921915* is also related with TG and TCHO, and *rs1800562* and *rs651007* are related with LDL-C and TCHO [28]. Nevertheless, removing the 4 SNPs (*rs174577*, *rs4921915*, *rs1800562* and *rs651007*) did not change the trends in the obtained results (Supplementary Figs. 4, 5, 6 and 7).

## Discussion

Previous traditional observational studies have shown inconsistent results regarding the relationship between iron status and breast cancer [7–11], likely owing to differences in race, ethnicity and sample sizes. Moreover, reverse causality and residual confusion may also be present in these studies, and unconsidered confounding factors or unknown risk factors may influence the observed correlations between iron status and breast cancer. To overcome these limitations, in this study, we conducted two-sample MR to estimate the causal relationship between iron status and breast cancer risk using summary statistics data from the largest meta-GWASs of the European population. Our results showed that serum transferrin levels were positively associated with the risk of ER-positive breast cancer, but that other iron statuses had no association with the risk of breast cancer or its subtypes. To the best of our knowledge, the present study is the first to use MR to investigate the association between iron status and breast cancer.

In the causal estimation of the relationship between serum transferrin and ER-negative breast cancer risk, we used the simple mode approach in addition to the three traditional methods. A positive correlation [OR, 1.225 (1.084, 1.366); *P* = 0.030] was observed between transferrin levels and ER-negative breast cancer risk. It has been speculated that a high concentration of transferrin and transferrin receptors could increase the transport efficiency of iron, increasing intracellular iron concentration. This increase in iron concentration could lead to lipid peroxidation, gene mutations, DNA strand breakage and oncogene activation, thus leading to an increased risk of breast cancer [29, 30].

In addition, the simple mode approach is a new method wherein the mode-based estimate can be used to obtain a single causal estimation from multiple genetic instruments. It provides robustness to horizontal pleiotropy in a different manner than the IVW, MR-Egger and weighted median methods. The simple mode provides better detection capability than the MR-Egger estimate, although its detection capability is inferior to that of the IVW and weighted median methods [17]. In addition, the causal estimates of the simple mode for the relationship between transferrin and ER-negative breast cancer were marginally significant; hence, this relationship needs to be studied further.

As there is some risk of pleotropic effects in MR estimates [31], we searched for the possibility of this secondary effect using SNPs from the PubMed database. Our online search identified 4 SNPs — *rs1800562* in *HFE*, *rs174577* in *FADS2*, *rs651007* in *ABO* and *rs4921915* in *NAT2* — related to LDL-C, TCHO, and/or TG levels, which have been reported to influence breast cancer risk [31, 32]. Nevertheless, the removal of these SNPs from our analysis did not cause any substantial changes in the MR estimation results, indicating that our results are unlikely to be biassed by blood lipid levels. In order to further test the robustness of our findings with regard to potential pleiotropic effects, we increased the number of SNPs available for analysis by relaxing the selection criteria for instrumental variables. In the sensitivity analysis, we found no significant differences via both the conservative and liberal approaches. The slight difference in estimation and confidence interval width between the different MR analysis methods may be accidental or a result of differences in measurement error, instead of reflecting actual differences. In addition, in the pleiotropic test, the MR-Egger method detected no bias. The public GWAS data on both exposure and outcome came from European cohorts, minimising the population bias. Further, our calculation results for the leave-one-out MR analysis were similar to the main MR estimates. Taken together, it appeared that there was no serious bias in the overall analysis and conclusion of our study.

Breast cancer is a heterogeneous disease with different histopathology and molecular subtypes, each with their own clinical prognosis and risk factors [33]. Previous studies have suggested that obesity may increase the risk of breast cancer [34]. Furthermore, BMI is related to iron concentrations and breast cancer risk [35, 36]. Data on BMI-related genetics were obtained from the GIANT consortium for 339,224 individuals of European descent [37]. None of the 11 SNPs we selected as instrumental variables were significantly associated with BMI (Table 5).

The change in iron status and the risk of breast cancer may be caused by common exposure factors. For example, inflammation affects iron status, increasing serum ferritin concentrations and reducing serum iron concentration [28, 38], and it could also increase oxidative tumour stress. Hence, inflammation may lead to an increase in iron status and the risk of breast cancer. However, due to the limited literature on this topic, further research is needed to fully explore these relationships.

There are some advantages to our study. We evaluated summary data from the largest meta-GWASs available, the GIS consortium and the OncoArray network. All the data extracted were from individuals of European descent, reducing any ancestry-related bias. In addition, we used two analysis methods to select instrumental variables — conservative and liberal analyses — which guarantees the robustness of our causal estimation.

However, there are some limitations to our present study. First, due to the limitations of the publicly available GWAS databases, it was difficult to perform hierarchical analysis according to factors such as age and sex in the combined exposures and outcome databases. Second, we used liberal instruments, which although provide more power to the study, also make the study particularly vulnerable to effects of pleiotropy. In this study, although we tried to reduce the effects of pleiotropy, bias due to the unknown biological functions of the SNPs concerned with iron status may be inevitable. Moreover, under ideal condition, for MR analysis, a sample size as large as possible should be used to make the results more reliable. Although the data for iron status and breast cancer were obtained from the GWAS databases with the largest sample size in the world, the sample size may still not have been fully ideal. The cohorts used in our study are all from European races, which contributing to reducing the bias brought by races. However, it is unknown whether the results are suitable for other races. Therefore, more researches should be conducted.

## Conclusion

Our MR study indicates that changes in the serum transferrin concentration could increase the risk of ER-negative breast cancer, whereas the status of the other three iron statuses had no association with breast cancer. As the liberal instrument was relatively weak, these findings need to be verified in further studies.

## Supplementary Information

**Additional file 1: Supplementary Fig. 1.** The SNP effects on iron status biomarkers and breast cancer for Scatterplot. iron (a), transferrin (b), ferritin (c), and transferrin saturation (d). MR Egger, Mendelian randomization–Egger regression method.

**Additional file 2: Supplementary Fig. 2.** The SNP effects on iron status biomarkers and ER-positive breast cancer for Scatterplot. iron (a), transferrin (b), ferritin (c), and transferrin saturation (d). MR Egger, Mendelian randomization–Egger regression method, ER, estrogen receptor.

**Additional file 3: Supplementary Fig. 3.** The SNP effects on iron status biomarkers and ER-negative breast cancer for Scatterplot. iron (a), transferrin (b), ferritin (c), and transferrin saturation (d). MR Egger, Mendelian randomization–Egger regression method; ER, estrogen receptor.

**Additional file 4: Supplementary Fig. 4.** Odds ratio of breast cancer, ER-positive breast cancer, and ER-negative breast cancer risk per standard deviation increase in iron excluding 1 SNP at per time estimated by the inverse variance weighted and Wald ratio (only for breast cancer). SNP: single nucleotide polymorphism; OR: odds ratio; 95% CI, 95% confidence interval; ER, estrogen receptor.

**Additional file 5: Supplementary Fig. 5.** Odds ratio of breast cancer, ER-positive breast cancer, and ER-negative breast cancer risk per standard deviation increase in transferrin excluding 1 SNP at per time estimated by the inverse variance weighted. SNP: single nucleotide polymorphism; OR: odds ratio; 95% CI, 95% confidence interval; ER, estrogen receptor.

**Additional file 6: Supplementary Fig. 6.** Odds ratio of breast cancer, ER-positive breast cancer, and ER-negative breast cancer risk per standard deviation increase in ferritin excluding 1 SNP at per time estimated by the inverse variance weighted. SNP: single nucleotide polymorphism; OR: odds ratio; 95% CI, 95% confidence interval; ER, estrogen receptor.

**Additional file 7: Supplementary Fig. 7.** Odds ratio of breast cancer, ER-positive breast cancer, and ER-negative breast cancer risk per standard deviation increase in Transferrin saturation excluding 1 SNP at per time estimated by the inverse variance weighted and Wald ratio (only for breast cancer). SNP: single nucleotide polymorphism; OR: odds ratio; 95% CI, 95% confidence interval; ER, estrogen receptor.

## Data Availability

OncoArray network https://epi.grants.cancer.gov/gameon/ GWAS catalogue database https://www.ebi.ac.uk/gwas GIANT consortium http://portals.broadinstitute.org/collaboration/giant/index.php/Main_Page

## Abbreviations

MR: Mendelian randomisation
SNPs: Single nucleotide polymorphisms
IVW: Inverse-variance weighted
GWAS: Genome-wide association study
GIS: Genetics of Iron Status
HWE: Hardy–Weinberg Equilibrium Test
MAF: Minor allele frequency
LDL-C: LDL cholesterol
HDL-C: HDL cholesterol
TG: Triglyceride
TCHO: Total cholesterol
MBE: Mode-based estimate
