# Supplementary material for: Serum iron status and the risk of breast cancer in the European population: a two-sample Mendelian randomisation study
Source: Genes Nutr. 2021 Jul 6;16:9. doi: 10.1186/s12263-021-00691-7 (PMC8259019; doi:10.1186/s12263-021-00691-7)
Supplement: Supplementary file 1 — Additional file 1: Supplementary Fig. 1. The SNP effects on iron status biomarkers and breast cancer for Scatterplot. iron (a), transferrin (b), ferritin (c), and transferrin saturation (d). MR Egger, Mendelian randomization–Egger regression method. [file 12263_2021_691_MOESM1_ESM.pdf]

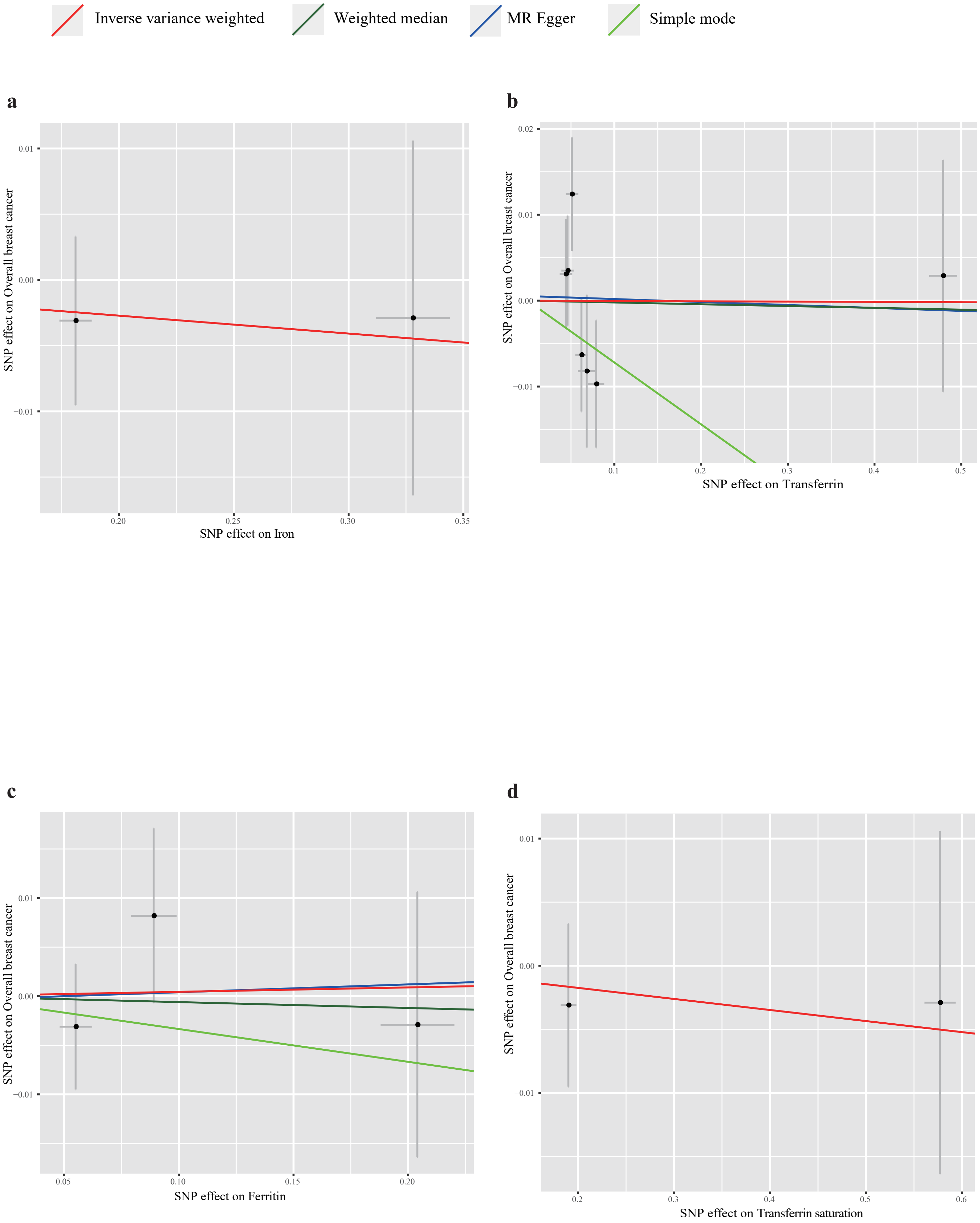

Supplementary Fig. 1 The SNP effects on iron status biomarkers and breast cancer for Scatterplot. iron (a), transferrin (b), ferritin (c), and transferrin saturation (d). MR Egger, Mendelian randomization–Egger regression method.
