# Supplementary material for: Serum iron status and the risk of breast cancer in the European population: a two-sample Mendelian randomisation study
Source: Genes Nutr. 2021 Jul 6;16:9. doi: 10.1186/s12263-021-00691-7 (PMC8259019; doi:10.1186/s12263-021-00691-7)
Supplement: Supplementary file 3 — Additional file 3: Supplementary Fig. 3. The SNP effects on iron status biomarkers and ER-negative breast cancer for Scatterplot. iron (a), transferrin (b), ferritin (c), and transferrin saturation (d). MR Egger, Mendelian randomization–Egger regression method; ER, estrogen receptor. [file 12263_2021_691_MOESM3_ESM.pdf]

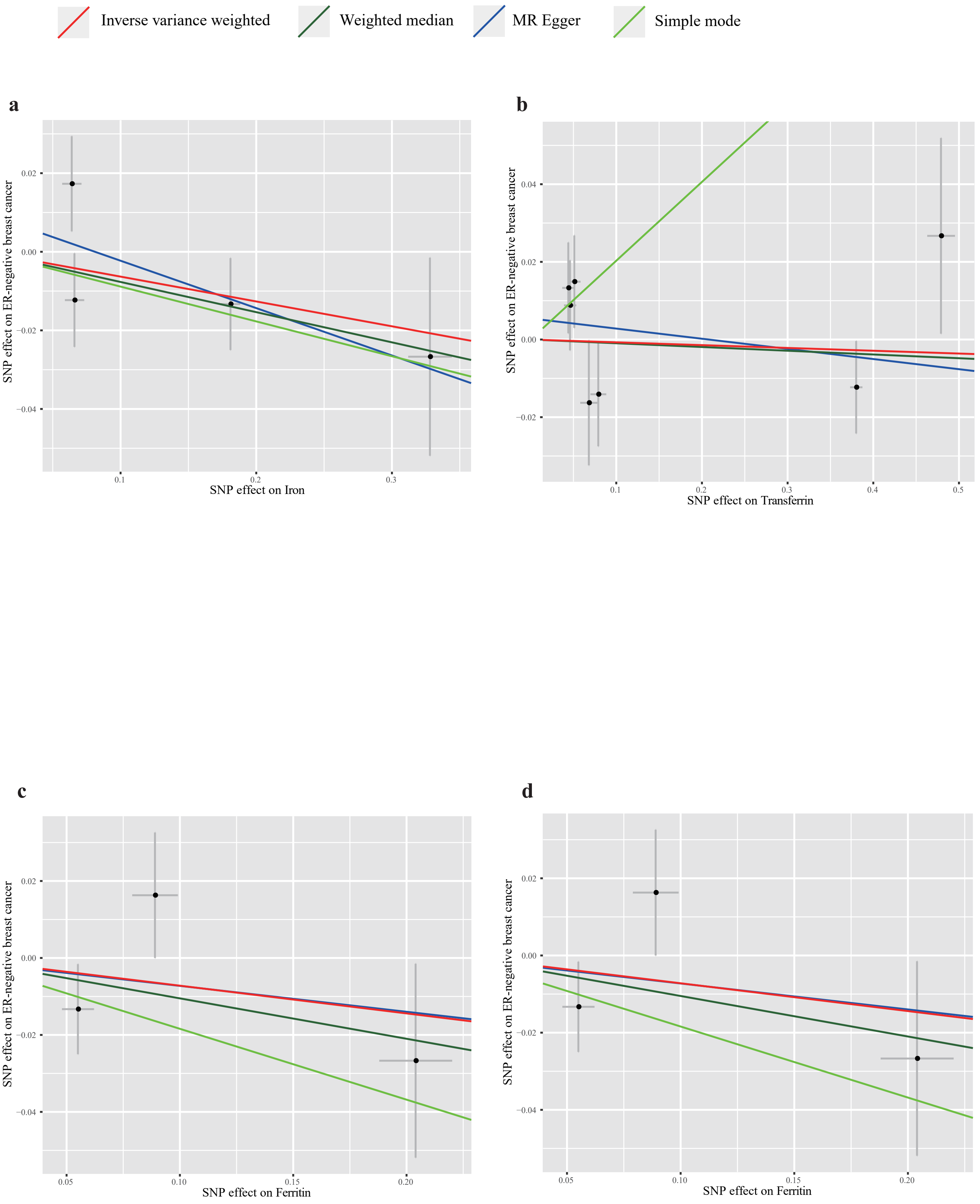

Supplementary Fig. 3 The SNP effects on iron status biomarkers and ER-negative breast cancer for Scatterplot. iron (a), transferrin (b), ferritin (c), and transferrin saturation (d). MR Egger, Mendelian randomization–Egger regression method; ER, estrogen receptor.
