# Supplementary material for: Serum iron status and the risk of breast cancer in the European population: a two-sample Mendelian randomisation study
Source: Genes Nutr. 2021 Jul 6;16:9. doi: 10.1186/s12263-021-00691-7 (PMC8259019; doi:10.1186/s12263-021-00691-7)
Supplement: Supplementary file 6 — Additional file 6: Supplementary Fig. 6. Odds ratio of breast cancer, ER-positive breast cancer, and ER-negative breast cancer risk per standard deviation increase in ferritin excluding 1 SNP at per time estimated by the inverse variance weighted. SNP: single nucleotide polymorphism; OR: odds ratio; 95% CI, 95% confidence interval; ER, estrogen receptor. [file 12263_2021_691_MOESM6_ESM.pdf]

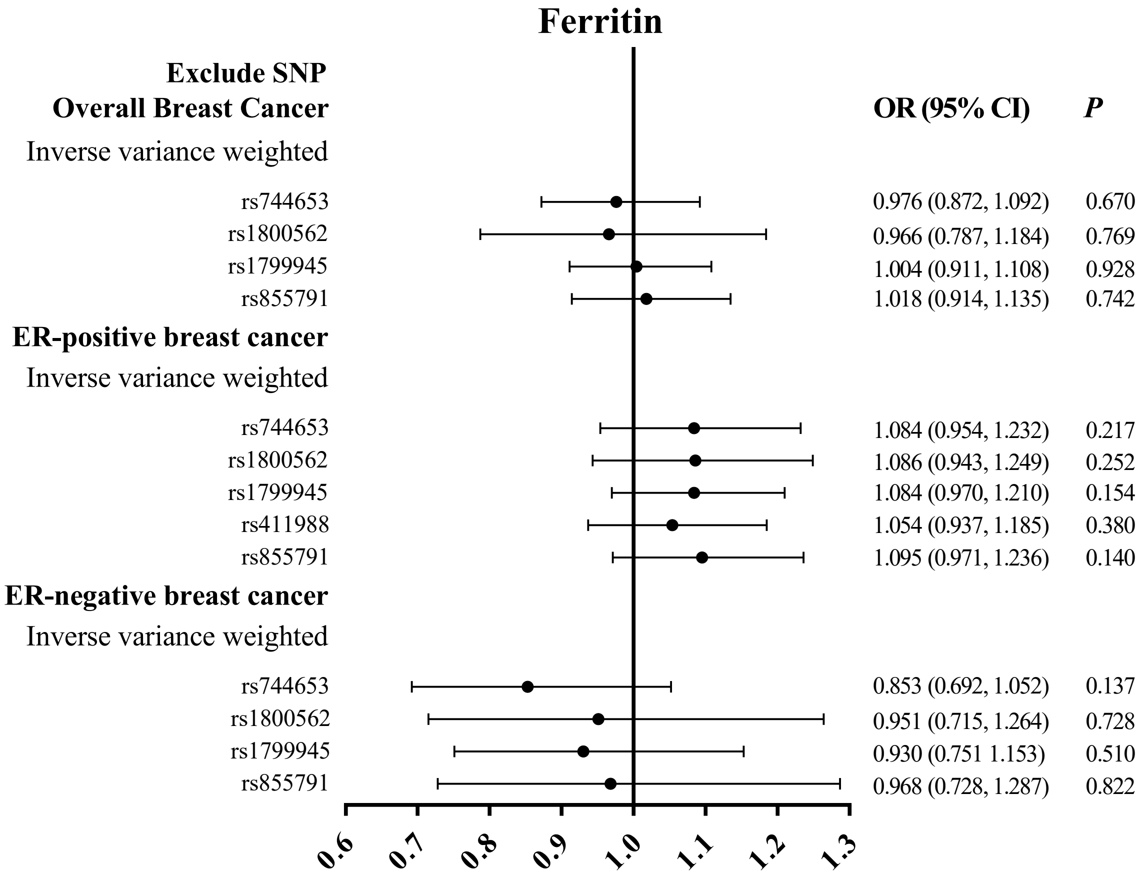

Supplementary Fig. 6 Odds ratio of breast cancer, ER-positive breast cancer, and ER-negative breast cancer risk per standard deviation increase in ferritin excluding 1 SNP at per time estimated by the inverse variance weighted. SNP: single nucleotide polymorphism; OR: odds ratio; 95% CI, 95% confidence interval; ER, estrogen receptor.
